# Correlates of condom use among male university students from eastern China who engage in casual sex

**DOI:** 10.1371/journal.pone.0283970

**Published:** 2023-05-25

**Authors:** Weiyong Chen, Xin Zhou, Qiaoqin Ma, Lin He, Wanjun Chen, Zhihong Guo, Lin Chen

**Affiliations:** Department of HIV/AIDS and STDs Control and Prevention, Zhejiang Provincial Center for Disease Control and Prevention, Hangzhou, Zhejiang, P. R. China; Anhui Agricultural University, CHINA

## Abstract

Consistent condom use with casual partners is critical for preventing the transmission of human immunodeficiency virus (HIV) among male university students. This study aimed to determine the level of consistent condom use and explore the correlates of condom use consistency in male university students in eastern China. A descriptive cross-sectional survey was conducted in 13 universities in Zhejiang Province, which involved the recruitment of 31,674 students by stratified random sampling. Among them, 545 male students who engaged in casual sex in the year prior to this study were included. Adjusted and unadjusted logistic regression models were used to examine the correlates associated with consistent condom use. Among the 545 male university students, only 205 (37.6%) consistently used condoms in the previous year. The following correlates were associated with higher rates of consistent condom use: 1) Knowledge, specifically, the number of correct answers to “HIV infection can be determined by appearance” (AOR: 2.06, 95% CI: 1.21–3.49); 2) never finding casual partners on the internet during the past over the prior year (AOR: 0.63; 95% CI: 0.40–0.99); 3) never drinking alcohol before casual sex during the last over the prior year (AOR: 0.30; 95% CI: 0.20–0.46); 4) never engaging in commercial sex (AOR: 0.57; 95% CI: 0.34–0.96); and 5) high condom self-efficacy score (AOR: 2.55; 95% CI: 1.44–4.49). The study found a low level of consistent condom use among male university students. Promoting condom self-efficacy, reducing web-based casual sex, drinking before sex, and commercial sex are essential to improving the level of consistent condom use among male university students to reduce the transmission of HIV.

## Introduction

Since the 1980s, the incidence of human immunodeficiency virus (HIV) transmission continues to increase worldwide. In 2014, the Joint United Nations Programme on HIV and acquired immunodeficiency syndrome (AIDS) (UNAIDS) proposed a global goal to end the AIDS epidemic by 2030 as a public health threat. However, the 2020 UNAIDS report showed a significant gap between the current status and ending goal [1]. By 2018, a total of 11–23 million teenagers were living with HIV worldwide; additionally, AIDS-related death was one of the main causes of death in this population [2, 3].

In China, a continued increase is observed in the national annual number of students diagnosed with HIV; specifically, the number increased from 794 to 3077 between 2010 and 2017, with >30% growth [4, 5]. Among them, 90% were male students, and 80% reported homosexual behavior [6]. Men who have sex with men (MSM) were at a high risk of contracting HIV infection. However, an increased risk of HIV infection was found in heterosexual male university students among high-risk populations. Some studies reported that male university students exhibited the increased sexual behavior rate compared with female university students in China [7, 8]. Additionally, the proportion of high-risk sexual behaviors, such as commercial sex, casual sex, and drinking alcohol before sex, continues to increase worldwide [9, 10]. Another issue should be considered: heterosexual college students were engaged in homosexual activities that significantly impacted HIV transmission [11].

Increasing high-risk sexual behaviors have led to increased sexually transmitted infections (STIs) and abortion rates in college students [12, 13]. Over the past decade, an annual increase has been observed regarding the number of 18–24-year-old HIV students. More than 3,000 students have been infected in China in the last two years.

Several studies found that HIV-relevant knowledge, causal sexual behaviors, condom use, and HIV intervention increased vulnerability to inconsistent condom use among homosexual college students [14, 15]. However, lack of studies identifying the correlates associated with condom use in heterosexual male college students who engaged in casual sex (refers to sex with multiple partners rather than regular partner and/or commercial partners). In China, several studies have assessed HIV-relevant knowledge, causal online sexual behaviors, substance abuse, and self-efficacy of condom use among heterosexual students [16, 17]. Therefore, accurated understanding of the characteristics of male students who engage in casual sex is critical for HIV prevention and more research is needed.

In 2018, Health Commission of Zhejiang Province and Department of Education of Zhejiang Province initiated a pilot program for HIV prevention among 13 universities in Zhejiang Province. The principal objective of this program was to explore an universal education and behavior intervention model in universities, to enhance the risk perception of HIV infection and self-protection and to practice protective sexual behavior among students for the prevention of HIV. The cross-sectional study was performed at the beginning of the program implementation. This study identified the status and correlates of insistent condom use among male university students who engaged in casual sex. This study aimed to provide scientific guidance for HIV/AIDS prevention among male students, especially high-risk male students in China.

## Materials and methods

### Study design

The cross-sectional study was conducted between October and November 2018 in Zhejiang province, China. The inclusion and exclusion criteria for participants were:(1) were aged 18 and older; (2) consented to participate in the study; (3) those who cannot obtain informed consent were exluded.

Among 107 universities in Zhejiang Province, 13 (12%) were included in this study. The 13 universities were distributed in all 11 cities in Zhejiang Province, three universities were located in Hangzhou which is the provincial capital city. Universities were recommended by the local Centers for Disease Control and Prevention (CDC).

The stratified cluster sampling method was used in the study. Three faculty members from each university used by random number table method. Each faculty members were categorized into four strata, from grade one to grade four. Classes in each grade was numbered and selected by the random number table method. The total number of students selected per faculty members and per grade were at least 200. Classes with exceedingly high or low sex ratios were replaced by another class. Six faculties of the research program in Zhejiang Provincial CDC was in charge of sampling. A pilot survey was conducted among a class of students in a university in Lishui city in August 2018.

### Data collection

Counseling teachers of the selected classes were in charge of data collection. The electronic questionnaire was designed as a two-dimensional code. All students were instructed to scan the two-dimensional code through the “WeChat” application during class meetings. The two-dimensional code was sent to out-of-school interns by their counseling teachers. Electronic informed consent was provided before beginning the survey. All participants received face-to-face or telephone training before questionnaire investigation. Students were instructed to complete the questionnaire online in a private space. The students allowed to leave the classroom 20 minutes after the survey beginning.

31,674 students participated in this survey.13,420 (42.4%) were male including freshmen, sophomores, juniors, and seniors accounted which were 32.1%, 28.6%, 27.1% and 12.2%, respectively. Among the 13,420 male students, 2,734 males reported with sexually active, of whom 2665 responded sexual partner encountered during the last year. Among the 2665 males, 583 participants reported having engaged in casual sex during the last year. However, 32 male students did not provide a response to the question “condom use with casual sex partners over the previous year.” Finally, 545 students were involved in the analysis.

### Measures

Final questionnaire included the demographic characteristics, HIV-relevant knowledge and training, attitudes and status of risky sexual behavior, risk perception for HIV infection and self-efficacy of condom use.

Consistent condom use was defined as “use condom every time when having casual sex over the last year”. Attitude toward casual sexual behavior: Participants were asked “Did you accept casual sexual behavior?”.

Based on network or dating applications, casual partners could be referred by internet.

HIV-relevant knowledge comprised two parts: HIV infection can be identified by appearance (knowledge 1), consistent condom use could prevent HIV transmission (knowledge 2).

Students Self-assessment in HIV infection risk: students carry out HIV infection risk self-assessment by software which were released by school.

The self-efficacy in condom use scale included three parts: 1) Are you confident to discuss condom use with your partner before sex? 2) Are you confident to prepare a condom before sex? 3) Are you confident in rejecting sex when your partner refuses to use condom? Five responses were in each part: ‘extremely confident,’ ‘very confident,’ ‘confident,’ ‘not confident,’ and ‘extremely not confident’ and could be assigned scores of 3, 2, 1, 0, and -1, respectively. For regression analysis, the scores were divided into three levels: ≤4, 5–8, and 9; the level of self-efficacy of condom use was graded from low to high. Inconsistent condom use was deemed “ever have sex intercourse with no condom.” The Cronbach’s alpha coefficient in this variable was 0.771.

### Statistical analysis

Statistical Product and Service Solution (SPSS) 20.0 was used to analyze the data. The demographic characteristics were analyzed descriptively. The chi-square test was used to compare the difference in consistent condom use among groups with different demographic characteristics, attitudes, risky sexual behavior, HIV knowledge, and intervention. Multivariate logistic regression analyses (Backward: LR) were used to identify the independent risk factors associated with consistent condom use with casual partners over the last year (1 = consistent condom use, 0 = inconsistent condom use). Variables in the model including all demographic characteristics variables and those with p<0.1 was determined by the Chi-square test. Missing data were not included in the analysis but including in the table. All tests were two-sided; statistical significance was set at p<0.05.

### Ethics approval and informed consent

The study was approved by the Ethics Committee of Zhejiang Provincial Center for Disease Control and Prevention (number 2018–036). All participants signed an electronic inform consent form.

## Results

### Condom use

Among the 545 male students with casual sex behavior, 62.4% (95% CI: 58.3%-66.5%) reported consistent condom use with casual partners over the last year.

### Demographic characteristics

The 545 students had a mean age of 20.1 years (range 18–28 years), with most sophomores and juniors. Moreover, 54.3% of the participants were from the countryside, and 72.5% from the local province. Most students reported having good or very good relationships with parents.

Rate of consistent condom use in freshmen, sophomores, juniors, and seniors were 60.8%, 68.5%, 61.9% and 62.3%, respectively (χ2 = 8.105, P = 0.044). Additionally, no significant difference was observed in rate of consistent condom use among different groups of age, residence, hometown, and relationship with parents by Chi-square test Table 1.

**Table 1:** Relationship between Sociodemographic characteristics and consistent condom use among 545 male students with casual sexual behavior.

Variables	Total (N, %)	consistent condom use (N, %)	χ^2^	*P* value
Age(years)			4.547	0.103
18-	175(32.1)	120(68.6)		
20–21	293(53.8)	172(58.7)		
≥22	77(14.1)	48(62.3)		
Grade			8.105	0.044
One	107(19.6)	65(60.8)		
Two	184(33.8)	126(68.5)		
Three	194(35.6)	120(61.9)		
Four	60(11.0)	29(48.3)		
Hometown			1.847	0.174
Local province	395(72.5)	253(64.1)		
Other provinces	149(27.3)	86(57.7)		
Missing	1	0		
Residence			0.004	0.952
Countryside	296(54.3)	185(62.5)		
Town/city	249(45.7)	155(62.3)		
Emotional relationship with parents			0.889	0.641
Very good	434(79.6)	275(63.4)		
good	78(14.3)	46(59.0)		
Bad	33(6.1)	19(57.6)		

### Attitudes toward risky sexual behavior and status

78.5% and 57.1% students indicated that one-night stand and commercial sex, respectively, were ‘acceptable.’ Additionally, 22.2% reported history of sexual activity with another man. Regarding different kinds of sexual behavior over the last year, 68.8% engaged in sex with regular partner, 59.5% found casual partners online, 49.5% consumed alcohol before engaging in casual sex, and 32.5% engaged in commercial sex Table 2.

**Table 2:** The difference in condom persistence among male students with different casual sexual behavior, self-efficacy of condom use and other HIV related behavior.

Variables	Total(N, %)	consistent condom use (N, %)	χ^2^	*P* values
Attitude toward casual sexual behaivor			0.184	0.668
Unacceptable/Unknown	117(21.5)	46(39.3)		
Acceptable	428(78.5)	159(37.2)		
Attitude toward commercial sexual behavior			3.849	0.050
Unacceptable/Unknown	234(42.9)	99(42.3)		
Acceptable	311(57.1)	106(34.1)		
Type of previous sex partners			5.004	0.025
Female	424(77.8)	170(40.1)		
Male/Both	121(22.2)	35(28.9)		
Ever engaging in sex with regular partner over the prior one year			12.678	0.002
No	159(29.2)	78(49.1)		
Yes	375(68.8)	124(33.1)		
Missing	11	3		
Finding casual partners on the internet over the prior one year			21.899	0.000
No	214(39.3)	105(49.1)		
Yes	324(59.5)	96(29.6)		
Missing	7	4		
Drinking alcohol before conducting casual sex over the prior one year			67.117	0.000
No	271(49.7)	148(54.6)		
Yes	270(49.5)	57(21.1)		
Missing	4	0		
Ever engaging in commercial sex			31.264	0.000
No	362(66.4)	166(45.9)		
Yes	177(32.5)	38(21.5)		
Missing	6	1		
Condom use with regular partner over the prior one year			161.060	0.000
Never	113(30.1)	10(8.9)		
sometimes/often	143(38.1)	21(14.7)		
Consistently	119(31.7)	93(78.2)		

Significantly lower rate of consistent condom use between groups in those engaging in sex with males/both (28.9% & 40.1%), ever engaging in sex with regular partners (33.1% & 49.1%), finding casual partners online (29.6% & 49.1%), consuming alcohol before casual sex (21.1% & 54.6%), and ever engaging in commercial sex (21.5% & 45.9%) were found by Chi-square test Table 2.

### Knowledge and training

Regarding the four HIV knowledge parts, the highest correct proportion were knowledge four (92.5%), knowledge one (77.6%), knowledge three (73.0%), and knowledge two (48.8%). Regarding the intervention in school over the last year, 70.8% reported receiving lectures on HIV/AIDS, 70.1% reported receiving health education on HIV testing, and 51.2% indicated accessing self-assessment implementation of HIV infection risk.

Percentage of consistent condom use in participants who answered HIV knowledges three (44.0%) and four (3.9%) correctly was significantly higher than that in those who answered incorrectly (20.4%) or unknown (9.8%) Table 3.

**Table 3:** The difference in condom persistence among male students with different HIV knowledge and intervention.

Variables	Total(N,%)	Consistent condom use(N, %)	χ^2^	*P* values
Knowledge 1			19.264	0.000
Incorrect/unknown	147(27.0)	30(20.4)		
Correct	398(73.0)	175(44.0)		
Knowledge 2			14.663	0.000
Incorrect/unknown	41(7.5)	4(9.8)		
Correct	504(92.5)	201(39.9)		
Received lectures on HIV/AIDS in school over the prior one year			0.152	0.670
No	159(29.2)	62(39.0)		
Yes	386(70.8)	143(37.1)		
Received propaganda on HIV testing in school over the prior one year			0.004	0.952
No	163(29.9)	61(37.4)		
Yes	382(70.1)	144(37.7)		
Self-assessment implement of HIV infection risk in school over the prior one year			3.095	0.079
No	266(48.8)	110(41.4)		
Yes	279(51.2)	95(34.1)		
Risk perception for HIV infection			16.659	0.000
No/Unknown	441(80.9)	184(41.7)		
Yes	98(18.0)	20(20.4)		
Missing	6	1		
Self-efficacy of condom use scale			8.289	0.040
0~4	120(22.0)	32(26.7)		
5~8	173(31.7)	71(41.0)		
9	248(45.5)	101(40.7)		
Missing	4	1		

### Risk perception for HIV infection and self-efficacy of condom use

All participants who responded to the questions, only 18.0% thought that they could possibly be infected by HIV. The self-efficacy of condom use. A total of 45.5% obtained a score of ≥9, 31.7% 5–8, and 22%, <4. Students who scored 5–8 and ≥9 reported higher percentage of consistent condom use than those who scored <4 (41.0% & 40.7% & 26.7%, χ^2^ = 8.289, *P* = -.040) Table 3.

### Results from the multivariable analysis

Results of the mixed-effects logistic regression models were showed in Table 4. Believing “It can identify an HIV patient by appearance” was associated with a higher likelihood of engaging in consistent condom use (AOR 2.06; 95% CI 1.21–3.49; p value = 0.007). Finding casual partners online over the last year was not significantly associated with a lower likelihood of engaging in consistent condom use (AOR 0.63; 95% CI 0.40–0.99; p value = 0.063). In contrast, “drinking alcohol before casual sex” was significantly associated with a lower likelihood of consistent condom use (AOR = 0.30; 95% CI 0.20~0.46; p value<0.0001). Ever engaging in commercial sex was negatively associated with consistent condom use (AOR: 0.57; 95% CI: 0.34–0.96; P value = 0.033). Regarding reported self-efficacy of condom use, students obtained scores of 5–8 (AOR 1.85; 95% CI 1.03~3.32, p = 0.040) and 9 (AOR: 2.55; 95% CI: 1.44–4.49; P = 0.001) were more likely to have consistent condom use than those who scored <4.

**Table 4:** Adjusted odds ratios from mixed effects logistic models examining association between consistent condom use and related predictors.

Variable	β	* $s_{\bar{x}}$ *	Wald χ^2^	*P* values	AOR(95%CI)
Knowledge 1					
Wrong/don’t know					1.00
Correct	0.722	0.270	7.163	0.007	2.06(1.21~3.49)
Finding casual partners on the internet over the prior one year					
No					1.00
Yes	-0.461	0.228	4.083	0.043	0.63(0.40~0.99)
Drinking alcohol before casual sex over the prior one year					
No					1.00
Yes	-1.202	0.220	29.800	0.000	0.30(0.20~0.46)
Ever engaging in commercial sex					
No					1.00
Yes	-0.561	0.263	4.557	0.033	0.57(0.34~0.96)
Self-efficacy of condom use scale					
0~4					1.00
5~8	0.613	0.299	4.200	0.040	1.85(1.03~3.32)
9	0.934	0.290	10.403	0.001	2.55(1.44~4.49)

## Discussion

This cross-sectional survey aimed to reflect the current situation of male university students condom use in casual sex in East China. Proportion of insistent condom use during casual sex between eastern China and other areas were regarded. such as proportion lower than United States [18–20]. China has traditional concept in sex, which emphasizes privacy between parents and children, between teachers and students. However, sexual education by some experts has gradually changed this concept in recent research. Nevertheless, school remains keep accessible place for students to learn about sex and sexual health.

Overall rate of HIV/AIDS knowledge among male university students who engage in casual sex was slightly lower compared to three universities in South China (89.1%) and higher compared to a university in North China (72.4%) were found in this study [21, 22]. Additionally, consistent condom use had a better overall rate of HIV/AIDS knowledge than those with inconsistent condom use among male students. Multivariate analysis showed that the knowledge that "appearance could identified whether a person is infected with AIDS" was a protective factor for male students who continued to use condoms with casual sex partners. Improving the knowledge of HIV/AIDs, promoting consistency of knowledge and action are essential to protect people in high risk behavior [23, 24]. In order to prevent the spread of HIV/AIDS in university students, 95% of university students should be knowledgeable about HIV/AIDS and continuous implementation of HIV/AIDS prevention programs need to be emphasised HIV-related knowledge, especially in HIV epidemic and key risk groups.

Male university students were more likely to engage in HIV high risk behaviors, such as commercial sex, multiple partners, sexual assault, and none condom use after drinking. Alcohol consumption is directly related to sexual assault. Alcohol were resulted in >64% of one-night stands in American college students [25–27]. Condoms are not consistently use in casual sex after drinking in this study, which is consistent with previous studies. However, drinking is a common behavior among university male students in China. Therefore, interventions to reduce alcohol abuse and safe sex after drinking should be strengthened in university.

Furthermore, inconsistent condom use was reported in male students who have casual sexual partners through the Internet or engaging in commercial sex. Previous studies have reported increased popularity in finding sexual partners conveniencely and anonymity through the Internet. However, it has been associated with HIV/STI transmission risk [28]. New challenge need to paid significant attention. Shortage of sexual education in university students in China, was results in unoptimistic problem on their sexual health [29, 30]. Innovative and optimized prevention and treatment methods in HIV/AIDS should be provided through internet communities, including preexposure and postexposure prophylaxis and self-testing services. Promotion on risky sexual behaivor and Individuals need to afford the responsibility in their own health should be established, especially university students [31, 32].

Some studies show that insistance in condom use was related self-efficacy expectations. Additionally, self-efficacy of condom use is the most important indicator affecting condom use [33–35]. In this study, self-efficacy in condom use was a significant factor among male students with casual sex. Furthermore, self-efficacy in condom use was different between male students with casual and consistent partners. Communication with casual sexual partners is more complex, which challenges the improvements of a person’s self-efficacy in condom use. Depression, attitude toward HIV/AIDS, and personality were influential factors in promoting self-efficacy incondom use in some studies [36, 37]. Therefore, further studies and interventions should be focused on finding these correlates for self-efficacy of condom use in male university students.

Several limitations were in this study. Firstly, it was a cross-sectional survey which the formulation of causal inference between influencing correlates and condom use. Secondly, discrepancy may be observed in behaviors of participants, due to they need to answer sensitive questions and recall bias might be existed for self-reported survy. Finally, this study was not specifically designed for male university students which lead to measurement weaknesses.

## Conclusion

The study revealed the proportion of risk behaviors among male university students with casual partners in eastern China. High self-efficacy of condom use was a protective factor for consistent condom use. Additionally, finding partners on the internet, drinking alcohol before sex, and engaging in commercial sex are high-risk correlates for consistent condom use. Interventions should focus on these correlates for promoting consistent condom use and avoiding detrimental effects among male university students caused by HIV.

## Supporting information

S1 Data (XLS)

## Abbreviations

AIDS: Acquired immunodeficiency syndrome
AOR: Adjusted odds ratio
CDC: Center for Disease Control and Prevention
CI: Confidential Interval
HIV: Human immunodeficiency virus
SPSS: Statistical Product and Service Solution
STI: Sexually transmitted infection
UNAIDS: the Joint United Nations Programme
